# Support for a Lower State Pension Age for Disadvantaged Older Workers

**DOI:** 10.1093/geroni/igaa057.1501

**Published:** 2020-12-16

**Authors:** Jaap Oude Mulders, Hendrik Van Dalen, Kène Henkens

**Affiliations:** 1 Netherlands Interdisciplinary Demographic Institute, The Hague, Netherlands; 2 Netherlands Interdisciplinary Demographic Institute, Den Haag, Netherlands; 3 NIDI, Den Haag, Netherlands

## Abstract

Due to policy reforms, early exit from the labor market has decreased substantially and people are participating in the labor market until much higher ages than before. As a result, there are increasingly many people that struggle to continue working until they can comfortably retire, for example due to chronic health conditions or having to provide informal care. A potential solution would be to grant earlier access to state pension benefits (such as Social Security) for disadvantaged older workers. While it is known that many people are supportive of such a policy, the question remains how much earlier access would be granted under which circumstances. Here, using a quasi-experimental vignette design (10,350 observations nested in 2,070 respondents), we study how much earlier Dutch people would like to grant access to disadvantaged older workers. Relevant characteristics of older workers that are judged are the age at which they started working, the level of physical strain in their job, whether they have chronic health conditions, and whether they provide informal care to a loved one. The result show that, on average, people would grant older workers with chronic muscoskeletal conditions or cardiovascular disease one year earlier access to the state pension than normal, while older workers that provide daily informal care would be granted 10 months earlier access. Cumulative disadvantage could lead to a maximum of three years earlier access to pension benefits. This study provides important insights into fairness considerations surrounding state pension provisions, and implications for practice will be discussed.

